# Textiloma in the leg

**DOI:** 10.4103/0019-5413.33689

**Published:** 2007

**Authors:** Amol C Patel, Govind S Kulkarni, Sunil G Kulkarni

**Affiliations:** Department of Orthopedics, Post Graduate Institute of Swashiyog Prathisthan, Extension Area, Miraj - 416 410, India

**Keywords:** Textiloma, foreign body, pseudotumor

## Abstract

Textiloma is defined as a tumor formed due to retained gauze. It is rarely reported in the musculoskeletal system. We are presenting a case with a soft tissue swelling over the lateral aspect of the lower third of the leg, come for implant removal of the distal tibia and fibular fracture. We removed the soft tissue mass enbloc thinking it to be a benign tumor. On cutting the mass on the operation table, a gauze piece encased by fibrous tissue was found. Textiloma can present as tumoral forms and can mimic as a pseudo-tumor.

Textiloma is defined as a tumor composed of cotton matrix surrounded by granulomatous reaction.^1^ The reported frequency of accidentally forgotten foreign bodies in surgery varies between 1 in 1000 to 1 in 10000 interventions.^2^ Most of the cases are reported in the abdomen and very few in the limbs.^3^ It can present as a pseudo-tumor. We hereby report a case of textiloma which was found after two years of the surgery.

## CASE REPORT

**Figure 1:**
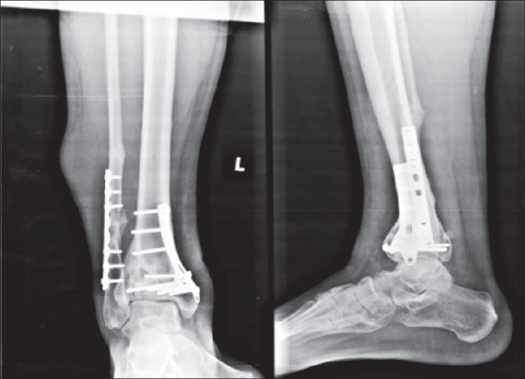
X-ray (AP and lateral view) of leg shows a soft tissue shadow on lateral aspect of leg

A 29-year-old man reported for implant removal of the distal end of the tibia. He had a symptomless swelling along the lateral aspect of the distal end of the leg. He was operated for distal end tibia fracture two years back by means of Ilizarov and fibular plate and again operated for tibial malunion by open reduction and plating one and half years back. The patient was having a normal gait. On examination a well-defined mass along the lateral aspect of the leg measuring 4 × 3 cm, oval to round shape, firm in consistency was found. There was no localized raised temperature, no abnormal vascularity. It was mobile painless and the skin over it was free and moving with the peroneal muscles. The surgical scar was overlying the mass. The laboratory investigation was normal. X-ray of the lower extremity and the ankle joint showed the soft tissue mass as a noncalcified tumefaction of the soft tissue adjacent to the fibular plate [Figure 1]. Surrounding bone was normal.

**Figure 2:**
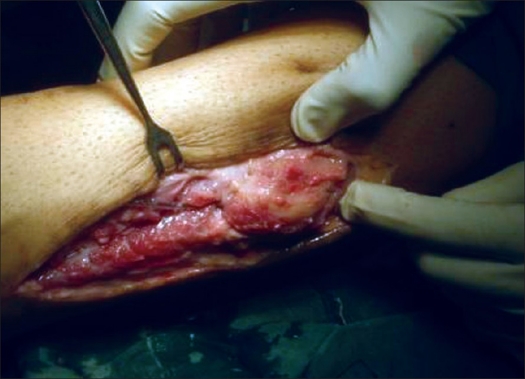
Intraoperative photograph shows well defined mass adjacent to fibular bone

Surgical excision along with the implant removal was planned. On surgery a well-defined mass was found in the peroneal brevis musculature with well-defined borders and adjacent to the fibular bone [Figure 2]. It did not have any attachments with the surrounding structures and could be enucleated.

**Figure 3:**
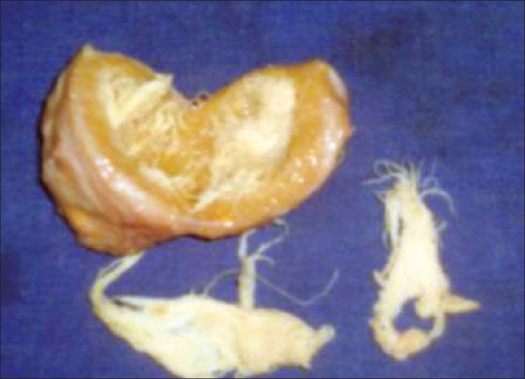
Clinical photograph of mass: On splitting the mass Gauze surrounded by fibrous tissue is seen

On cutting the lesion on the operation table, gauze surrounded by the fibrous tissue was noted [Figure 3]. Histological examination revealed a large foreign body granuloma. There was no sign of malignancy.

## DISCUSSION

A retained gauze is called textiloma. Glossypiboma (from Latin ‘gossipium’ - cotton and from Kiswahili ‘boma’- place of concealment)^4^ is an iatrogenic mass lesion caused by retained sponge. Both cause foreign body reaction.

Clinical picture of the textiloma may be acute (exudative) or delayed (tumoral). The exudative form usually presents with early clinical manifestation because of possibility of secondary super infection resulting in sepsis or because of formation of the fistula. It can be diagnosed with clinical suspicion. The tumoral forms appear late and may remain latent for many years.^5^

On X-ray it may show a soft tissue shadow or if calcified may show a whorl-like pattern. Radio marker can be seen if present.^6^ On ultrasongraphy it shows echogenic shadow with acoustic shadow. CT shows hypodense mass with thick peripheral rim. MR imaging reveals a well-defined mass lesion that shows intermediate signal intensity at T1-weighted imaging (T1WI) and slightly high signal intensity at T2-weighted imaging (T2WI). Wavy, low-signal-intensity stripes visible within the fluid-filled central cavity, is characteristic.^7^

Textiloma as a complication can occur in all forms of surgery. These are also seldom reported due to medicolegal problems. Only 6% of Textiloma are reported following orthopedic and spine surgery in comparison to 52% following abdominal surgery. No fatal complications have been noted in the muscular skeletal sites.^8^

The best approach to prevent textilomas is by simple gauze counting. Eleven cases of retained sponge are reported in one series where eight had presumed incorrect sponge count.^9^ Radio-opaque sponges can be used to identify them postoperatively and avoid future problems. But morbid mishap still may occur. It is important to note that the human errors cannot be completely abolished but continuous medical training and strict adherence to regulations should reduce the incidence to a minimum.^9^ Reactive, foreign-body-induced change may mimic bone and or soft tissue malignancies.^10^
